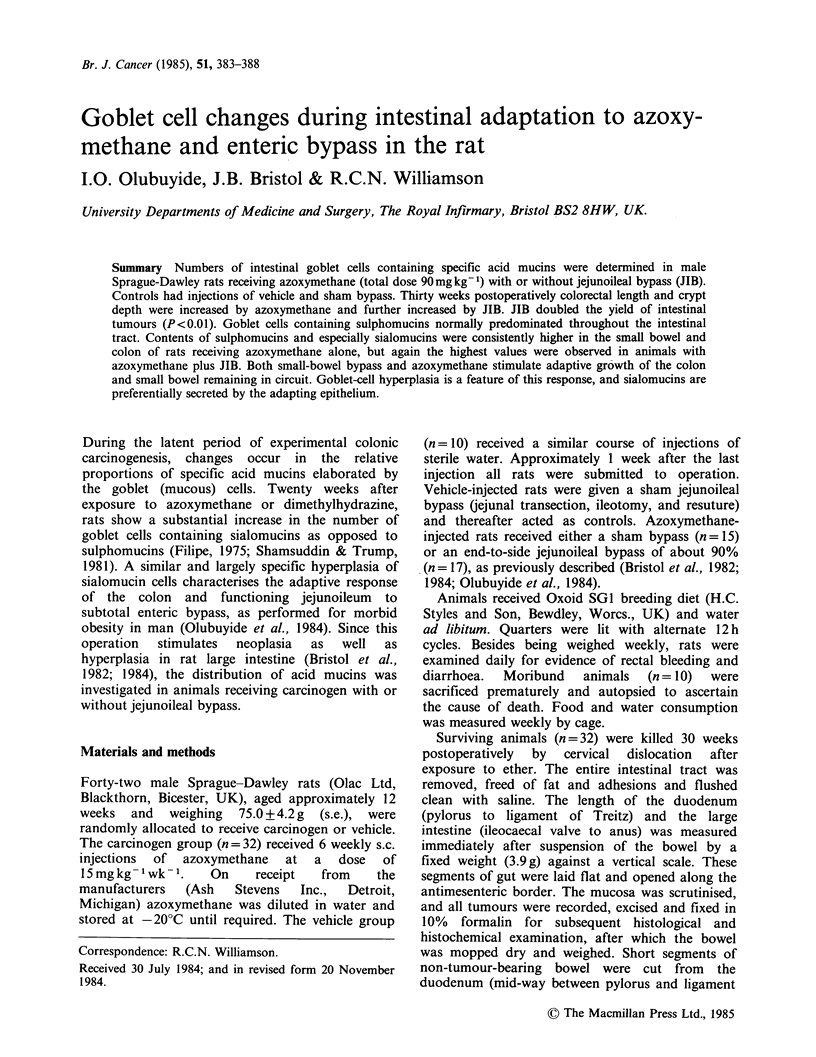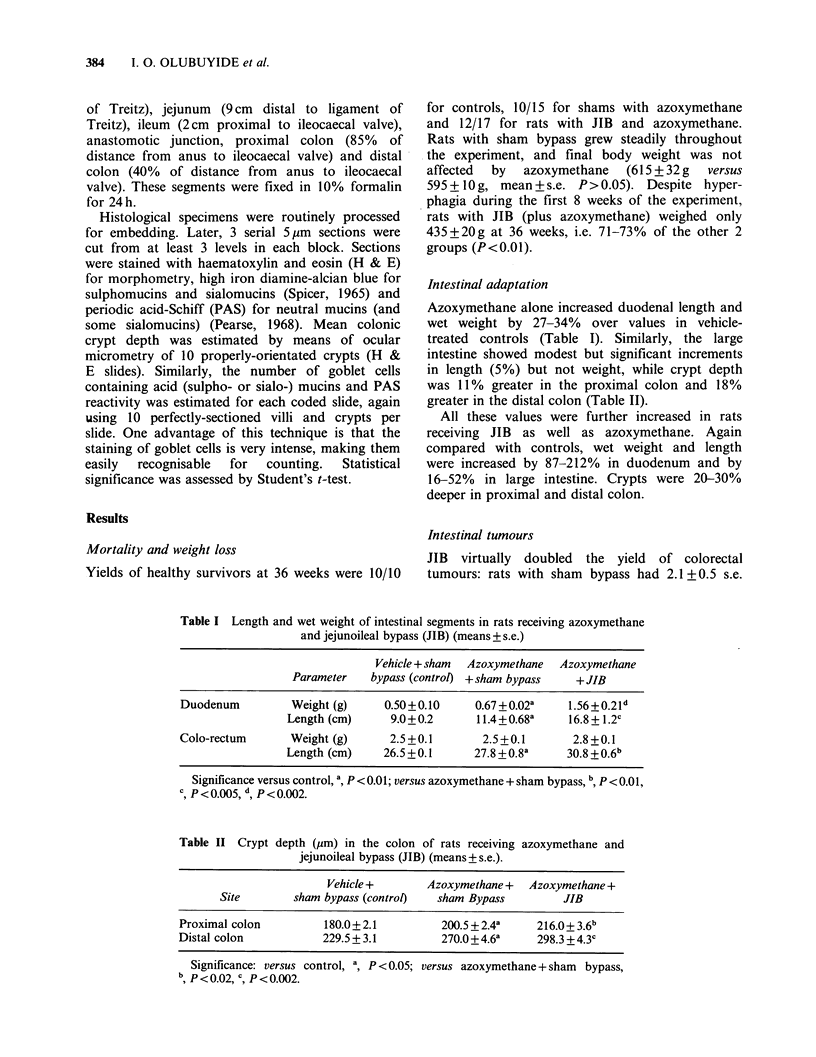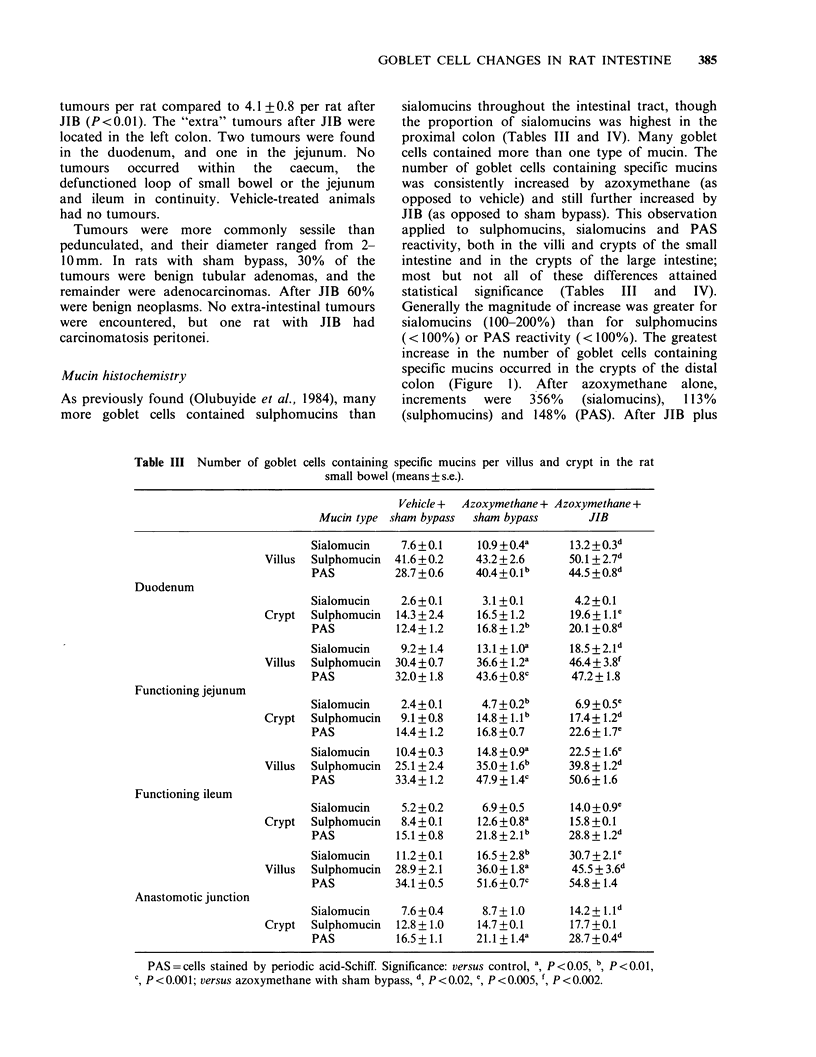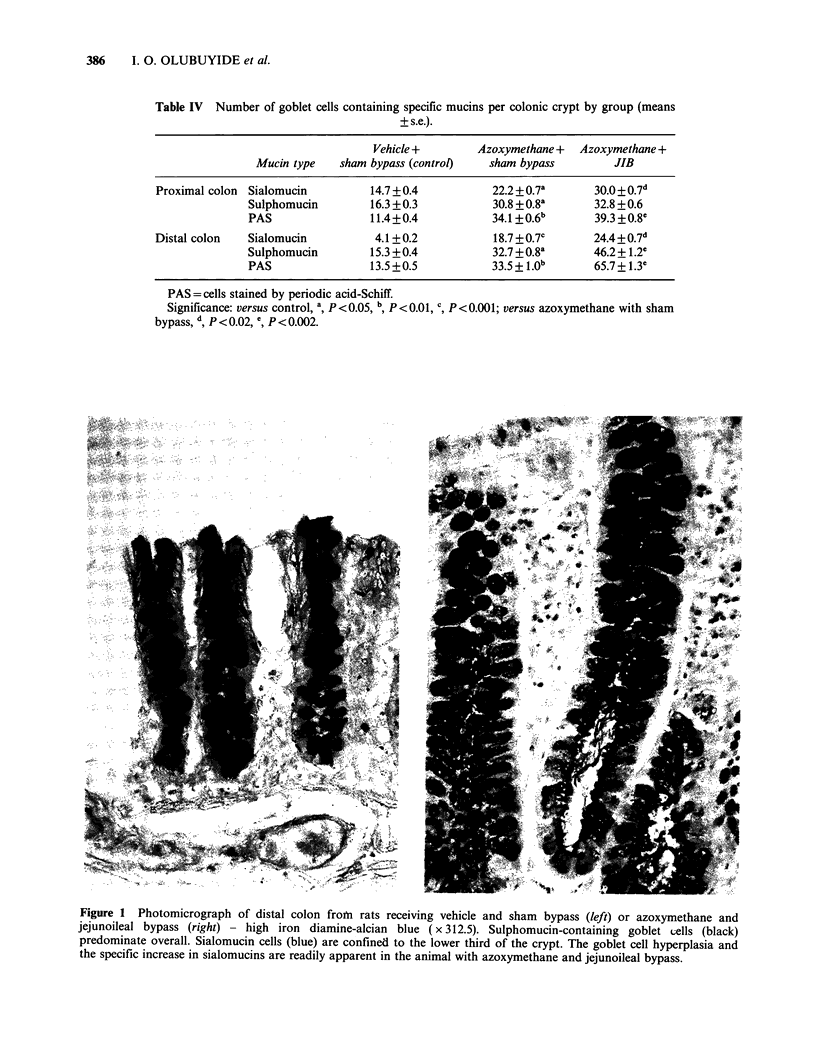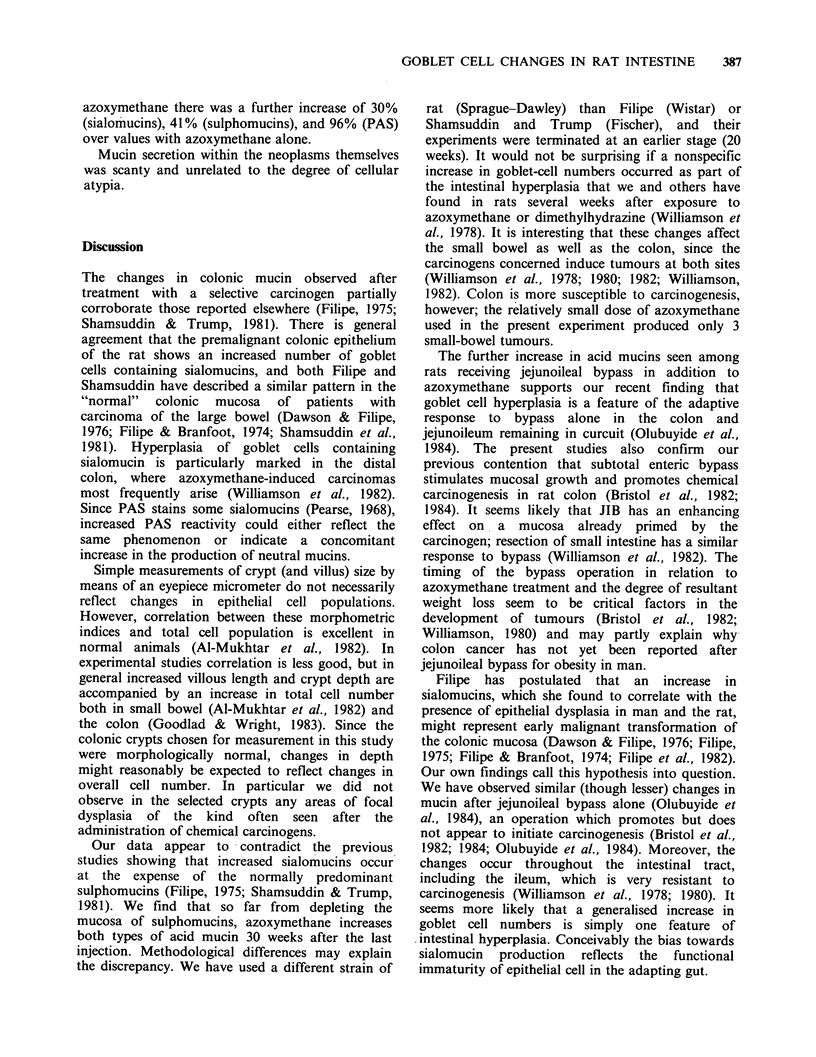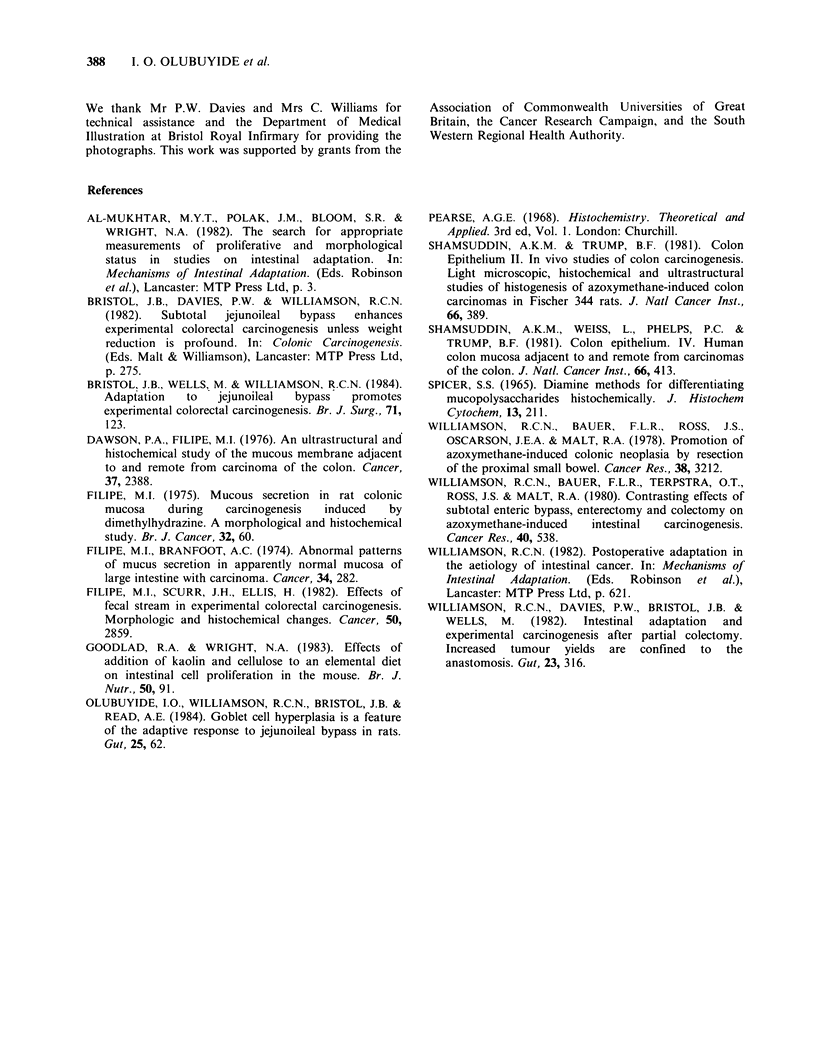# Goblet cell changes during intestinal adaptation to azoxymethane and enteric bypass in the rat.

**DOI:** 10.1038/bjc.1985.51

**Published:** 1985-03

**Authors:** I. O. Olubuyide, J. B. Bristol, R. C. Williamson

## Abstract

**Images:**


*Br. J. Cancer* (1985), **51**, 383–388

### Goblet cell changes during intestinal adaptation to azoxymethane and enteric bypass in the rat

I.O. Olubuyide, J.B. Bristol & R.C.N. Williamson

*University Departments of Medicine and Surgery, The Royal Infirmary, Bristol BS2 8HW, UK.*

**Summary** Numbers of intestinal goblet cells containing specific acid mucins were determined in male Sprague-Dawley rats receiving azoxymethane (total dose $90\,mg\,kg^{-1}$) with or without jejunoileal bypass (JIB). Controls had injections of vehicle and sham bypass. Thirty weeks postoperatively colorectal length and crypt depth were increased by azoxymethane and further increased by JIB. JIB doubled the yield of intestinal tumours ($P<0.01$). Goblet cells containing sulphomucins normally predominated throughout the intestinal tract. Contents of sulphomucins and especially sialomucins were consistently higher in the small bowel and colon of rats receiving azoxymethane alone, but again the highest values were observed in animals with azoxymethane plus JIB. Both small-bowel bypass and azoxymethane stimulate adaptive growth of the colon and small bowel remaining in circuit. Goblet-cell hyperplasia is a feature of this response, and sialomucins are preferentially secreted by the adapting epithelium.

During the latent period of experimental colonic carcinogenesis, changes occur in the relative proportions of specific acid mucins elaborated by the goblet (mucous) cells. Twenty weeks after exposure to azoxymethane or dimethylhydrazine, rats show a substantial increase in the number of goblet cells containing sialomucins as opposed to sulphomucins (Filipe, 1975; Shamsuddin & Trump, 1981). A similar and largely specific hyperplasia of sialomucin cells characterises the adaptive response of the colon and functioning jejunoileum to subtotal enteric bypass, as performed for morbid obesity in man (Olubuyide *et al.*, 1984). Since this operation stimulates neoplasia as well as hyperplasia in rat large intestine (Bristol *et al.*, 1982; 1984), the distribution of acid mucins was investigated in animals receiving carcinogen with or without jejunoileal bypass.

### Materials and methods

Forty-two male Sprague–Dawley rats (Olac Ltd, Blackthorn, Bicester, UK), aged approximately 12 weeks and weighing $75.0 \pm 4.2$ g (s.e.), were randomly allocated to receive carcinogen or vehicle. The carcinogen group ($n=32$) received 6 weekly s.c. injections of azoxymethane at a dose of $15\,mg\,kg^{-1}\,wk^{-1}$. On receipt from the manufacturers (Ash Stevens Inc., Detroit, Michigan) azoxymethane was diluted in water and stored at $-20$°C until required. The vehicle group ($n=10$) received a similar course of injections of sterile water. Approximately 1 week after the last injection all rats were submitted to operation. Vehicle-injected rats were given a sham jejunoileal bypass (jejunal transection, ileotomy, and resuture) and thereafter acted as controls. Azoxymethane-injected rats received either a sham bypass ($n=15$) or an end-to-side jejunoileal bypass of about 90% ($n=17$), as previously described (Bristol *et al.*, 1982; 1984; Olubuyide *et al.*, 1984).

Animals received Oxoid SG1 breeding diet (H.C. Styles and Son, Bewdley, Worcs., UK) and water *ad libitum*. Quarters were lit with alternate 12 h cycles. Besides being weighed weekly, rats were examined daily for evidence of rectal bleeding and diarrhoea. Moribund animals ($n=10$) were sacrificed prematurely and autopsied to ascertain the cause of death. Food and water consumption was measured weekly by cage.

Surviving animals ($n=32$) were killed 30 weeks postoperatively by cervical dislocation after exposure to ether. The entire intestinal tract was removed, freed of fat and adhesions and flushed clean with saline. The length of the duodenum (pylorus to ligament of Treitz) and the large intestine (ileocaecal valve to anus) was measured immediately after suspension of the bowel by a fixed weight (3.9 g) against a vertical scale. These segments of gut were laid flat and opened along the antimesenteric border. The mucosa was scrutinised, and all tumours were recorded, excised and fixed in 10% formalin for subsequent histological and histochemical examination, after which the bowel was mopped dry and weighed. Short segments of non-tumour-bearing bowel were cut from the duodenum (mid-way between pylorus and ligament

Correspondence: R.C.N. Williamson.

Received 30 July 1984; and in revised form 20 November 1984.

© The Macmillan Press Ltd., 1985

of Treitz), jejunum (9 cm distal to ligament of Treitz), ileum (2 cm proximal to ileocaecal valve), anastomotic junction, proximal colon (85% of distance from anus to ileocaecal valve) and distal colon (40% of distance from anus to ileocaecal valve). These segments were fixed in 10% formalin for 24 h.

Histological specimens were routinely processed for embedding. Later, 3 serial 5 $\mu$m sections were cut from at least 3 levels in each block. Sections were stained with haematoxylin and eosin (H & E) for morphometry, high iron diamine-alcian blue for sulphomucins and sialomucins (Spicer, 1965) and periodic acid-Schiff (PAS) for neutral mucins (and some sialomucins) (Pearse, 1968). Mean colonic crypt depth was estimated by means of ocular micrometry of 10 properly-orientated crypts (H & E slides). Similarly, the number of goblet cells containing acid (sulpho- or sialo-) mucins and PAS reactivity was estimated for each coded slide, again using 10 perfectly-sectioned villi and crypts per slide. One advantage of this technique is that the staining of goblet cells is very intense, making them easily recognisable for counting. Statistical significance was assessed by Student's *t*-test.

### Results

### *Mortality and weight loss*

Yields of healthy survivors at 36 weeks were 10/10 for controls, 10/15 for shams with azoxymethane and 12/17 for rats with JIB and azoxymethane. Rats with sham bypass grew steadily throughout the experiment, and final body weight was not affected by azoxymethane (615 ± 32 g *versus* 595 ± 10 g, mean ± s.e. $P > 0.05$). Despite hyperphagia during the first 8 weeks of the experiment, rats with JIB (plus azoxymethane) weighed only 435 ± 20 g at 36 weeks, i.e. 71–73% of the other 2 groups ($P < 0.01$).

### *Intestinal adaptation*

Azoxymethane alone increased duodenal length and wet weight by 27–34% over values in vehicle-treated controls (Table I). Similarly, the large intestine showed modest but significant increments in length (5%) but not weight, while crypt depth was 11% greater in the proximal colon and 18% greater in the distal colon (Table II).

All these values were further increased in rats receiving JIB as well as azoxymethane. Again compared with controls, wet weight and length were increased by 87–212% in duodenum and by 16–52% in large intestine. Crypts were 20–30% deeper in proximal and distal colon.

### *Intestinal tumours*

JIB virtually doubled the yield of colorectal tumours: rats with sham bypass had 2.1 ± 0.5 s.e.

**Table I** Length and wet weight of intestinal segments in rats receiving azoxymethane and jejunoileal bypass (JIB) (means ± s.e.)

| | *Parameter* | *Vehicle + sham bypass (control)* | *Azoxymethane + sham bypass* | *Azoxymethane + JIB* |
|---|---|---|---|---|
| Duodenum | Weight (g) | 0.50 ± 0.10 | 0.67 ± 0.02[a] | 1.56 ± 0.21[d] |
| | Length (cm) | 9.0 ± 0.2 | 11.4 ± 0.68[a] | 16.8 ± 1.2[c] |
| Colo-rectum | Weight (g) | 2.5 ± 0.1 | 2.5 ± 0.1 | 2.8 ± 0.1 |
| | Length (cm) | 26.5 ± 0.1 | 27.8 ± 0.8[a] | 30.8 ± 0.6[b] |

Significance versus control, [a], $P < 0.01$; *versus* azoxymethane + sham bypass, [b], $P < 0.01$, [c], $P < 0.005$, [d], $P < 0.002$.

**Table II** Crypt depth ($\mu$m) in the colon of rats receiving azoxymethane and jejunoileal bypass (JIB) (means ± s.e.).

| *Site* | *Vehicle + sham bypass (control)* | *Azoxymethane + sham Bypass* | *Azoxymethane + JIB* |
|---|---|---|---|
| Proximal colon | 180.0 ± 2.1 | 200.5 ± 2.4[a] | 216.0 ± 3.6[b] |
| Distal colon | 229.5 ± 3.1 | 270.0 ± 4.6[a] | 298.3 ± 4.3[c] |

Significance: *versus* control, [a], $P < 0.05$; *versus* azoxymethane + sham bypass, [b], $P < 0.02$, [c], $P < 0.002$.

tumours per rat compared to 4.1 ± 0.8 per rat after JIB ($P<0.01$). The "extra" tumours after JIB were located in the left colon. Two tumours were found in the duodenum, and one in the jejunum. No tumours occurred within the caecum, the defunctioned loop of small bowel or the jejunum and ileum in continuity. Vehicle-treated animals had no tumours.

Tumours were more commonly sessile than pedunculated, and their diameter ranged from 2–10 mm. In rats with sham bypass, 30% of the tumours were benign tubular adenomas, and the remainder were adenocarcinomas. After JIB 60% were benign neoplasms. No extra-intestinal tumours were encountered, but one rat with JIB had carcinomatosis peritonei.

*Mucin histochemistry*

As previously found (Olubuyide *et al.*, 1984), many more goblet cells contained sulphomucins than sialomucins throughout the intestinal tract, though the proportion of sialomucins was highest in the proximal colon (Tables III and IV). Many goblet cells contained more than one type of mucin. The number of goblet cells containing specific mucins was consistently increased by azoxymethane (as opposed to vehicle) and still further increased by JIB (as opposed to sham bypass). This observation applied to sulphomucins, sialomucins and PAS reactivity, both in the villi and crypts of the small intestine and in the crypts of the large intestine; most but not all of these differences attained statistical significance (Tables III and IV). Generally the magnitude of increase was greater for sialomucins (100–200%) than for sulphomucins (< 100%) or PAS reactivity (< 100%). The greatest increase in the number of goblet cells containing specific mucins occurred in the crypts of the distal colon (Figure 1). After azoxymethane alone, increments were 356% (sialomucins), 113% (sulphomucins) and 148% (PAS). After JIB plus

**Table III** Number of goblet cells containing specific mucins per villus and crypt in the rat small bowel (means ± s.e.).

| | | Mucin type | *Vehicle + sham bypass* | *Azoxymethane + sham bypass* | *Azoxymethane + JIB* |
|---|---|---|---|---|---|
| Duodenum | Villus | Sialomucin | 7.6 ± 0.1 | 10.9 ± 0.4[a] | 13.2 ± 0.3[d] |
| | | Sulphomucin | 41.6 ± 0.2 | 43.2 ± 2.6 | 50.1 ± 2.7[d] |
| | | PAS | 28.7 ± 0.6 | 40.4 ± 0.1[b] | 44.5 ± 0.8[d] |
| | Crypt | Sialomucin | 2.6 ± 0.1 | 3.1 ± 0.1 | 4.2 ± 0.1 |
| | | Sulphomucin | 14.3 ± 2.4 | 16.5 ± 1.2 | 19.6 ± 1.1[e] |
| | | PAS | 12.4 ± 1.2 | 16.8 ± 1.2[b] | 20.1 ± 0.8[d] |
| Functioning jejunum | Villus | Sialomucin | 9.2 ± 1.4 | 13.1 ± 1.0[a] | 18.5 ± 2.1[d] |
| | | Sulphomucin | 30.4 ± 0.7 | 36.6 ± 1.2[a] | 46.4 ± 3.8[f] |
| | | PAS | 32.0 ± 1.8 | 43.6 ± 0.8[c] | 47.2 ± 1.8 |
| | Crypt | Sialomucin | 2.4 ± 0.1 | 4.7 ± 0.2[b] | 6.9 ± 0.5[e] |
| | | Sulphomucin | 9.1 ± 0.8 | 14.8 ± 1.1[b] | 17.4 ± 1.2[d] |
| | | PAS | 14.4 ± 1.2 | 16.8 ± 0.7 | 22.6 ± 1.7[e] |
| Functioning ileum | Villus | Sialomucin | 10.4 ± 0.3 | 14.8 ± 0.9[a] | 22.5 ± 1.6[e] |
| | | Sulphomucin | 25.1 ± 2.4 | 35.0 ± 1.6[b] | 39.8 ± 1.2[d] |
| | | PAS | 33.4 ± 1.2 | 47.9 ± 1.4[c] | 50.6 ± 1.6 |
| | Crypt | Sialomucin | 5.2 ± 0.2 | 6.9 ± 0.5 | 14.0 ± 0.9[e] |
| | | Sulphomucin | 8.4 ± 0.1 | 12.6 ± 0.8[a] | 15.8 ± 0.1 |
| | | PAS | 15.1 ± 0.8 | 21.8 ± 2.1[b] | 28.8 ± 1.2[d] |
| Anastomotic junction | Villus | Sialomucin | 11.2 ± 0.1 | 16.5 ± 2.8[b] | 30.7 ± 2.1[e] |
| | | Sulphomucin | 28.9 ± 2.1 | 36.0 ± 1.8[a] | 45.5 ± 3.6[d] |
| | | PAS | 34.1 ± 0.5 | 51.6 ± 0.7[c] | 54.8 ± 1.4 |
| | Crypt | Sialomucin | 7.6 ± 0.4 | 8.7 ± 1.0 | 14.2 ± 1.1[d] |
| | | Sulphomucin | 12.8 ± 1.0 | 14.7 ± 0.1 | 17.7 ± 0.1 |
| | | PAS | 16.5 ± 1.1 | 21.1 ± 1.4[a] | 28.7 ± 0.4[d] |

PAS = cells stained by periodic acid-Schiff. Significance: *versus* control, [a], $P<0.05$, [b], $P<0.01$, [c], $P<0.001$; *versus* azoxymethane with sham bypass, [d], $P<0.02$, [e], $P<0.005$, [f], $P<0.002$.

**Table IV** Number of goblet cells containing specific mucins per colonic crypt by group (means ±s.e.).

| | *Mucin type* | *Vehicle + sham bypass (control)* | *Azoxymethane + sham bypass* | *Azoxymethane + JIB* |
|---|---|---|---|---|
| Proximal colon | Sialomucin | 14.7±0.4 | 22.2±0.7[a] | 30.0±0.7[d] |
| | Sulphomucin | 16.3±0.3 | 30.8±0.8[a] | 32.8±0.6 |
| | PAS | 11.4±0.4 | 34.1±0.6[b] | 39.3±0.8[e] |
| Distal colon | Sialomucin | 4.1±0.2 | 18.7±0.7[c] | 24.4±0.7[d] |
| | Sulphomucin | 15.3±0.4 | 32.7±0.8[a] | 46.2±1.2[e] |
| | PAS | 13.5±0.5 | 33.5±1.0[b] | 65.7±1.3[e] |

PAS = cells stained by periodic acid-Schiff.

Significance: *versus* control, [a], $P<0.05$, [b], $P<0.01$, [c], $P<0.001$; *versus* azoxymethane with sham bypass, [d], $P<0.02$, [e], $P<0.002$.

**Figure 1** Photomicrograph of distal colon from rats receiving vehicle and sham bypass (*left*) or azoxymethane and jejunoileal bypass (*right*) – high iron diamine-alcian blue (×312.5). Sulphomucin-containing goblet cells (black) predominate overall. Sialomucin cells (blue) are confined to the lower third of the crypt. The goblet cell hyperplasia and the specific increase in sialomucins are readily apparent in the animal with azoxymethane and jejunoileal bypass.

azoxymethane there was a further increase of 30% (sialomucins), 41% (sulphomucins), and 96% (PAS) over values with azoxymethane alone.

Mucin secretion within the neoplasms themselves was scanty and unrelated to the degree of cellular atypia.

### Discussion

The changes in colonic mucin observed after treatment with a selective carcinogen partially corroborate those reported elsewhere (Filipe, 1975; Shamsuddin & Trump, 1981). There is general agreement that the premalignant colonic epithelium of the rat shows an increased number of goblet cells containing sialomucins, and both Filipe and Shamsuddin have described a similar pattern in the "normal" colonic mucosa of patients with carcinoma of the large bowel (Dawson & Filipe, 1976; Filipe & Branfoot, 1974; Shamsuddin *et al.*, 1981). Hyperplasia of goblet cells containing sialomucin is particularly marked in the distal colon, where azoxymethane-induced carcinomas most frequently arise (Williamson *et al.*, 1982). Since PAS stains some sialomucins (Pearse, 1968), increased PAS reactivity could either reflect the same phenomenon or indicate a concomitant increase in the production of neutral mucins.

Simple measurements of crypt (and villus) size by means of an eyepiece micrometer do not necessarily reflect changes in epithelial cell populations. However, correlation between these morphometric indices and total cell population is excellent in normal animals (Al-Mukhtar *et al.*, 1982). In experimental studies correlation is less good, but in general increased villous length and crypt depth are accompanied by an increase in total cell number both in small bowel (Al-Mukhtar *et al.*, 1982) and the colon (Goodlad & Wright, 1983). Since the colonic crypts chosen for measurement in this study were morphologically normal, changes in depth might reasonably be expected to reflect changes in overall cell number. In particular we did not observe in the selected crypts any areas of focal dysplasia of the kind often seen after the administration of chemical carcinogens.

Our data appear to contradict the previous studies showing that increased sialomucins occur at the expense of the normally predominant sulphomucins (Filipe, 1975; Shamsuddin & Trump, 1981). We find that so far from depleting the mucosa of sulphomucins, azoxymethane increases both types of acid mucin 30 weeks after the last injection. Methodological differences may explain the discrepancy. We have used a different strain of rat (Sprague–Dawley) than Filipe (Wistar) or Shamsuddin and Trump (Fischer), and their experiments were terminated at an earlier stage (20 weeks). It would not be surprising if a nonspecific increase in goblet-cell numbers occurred as part of the intestinal hyperplasia that we and others have found in rats several weeks after exposure to azoxymethane or dimethylhydrazine (Williamson *et al.*, 1978). It is interesting that these changes affect the small bowel as well as the colon, since the carcinogens concerned induce tumours at both sites (Williamson *et al.*, 1978; 1980; 1982; Williamson, 1982). Colon is more susceptible to carcinogenesis, however; the relatively small dose of azoxymethane used in the present experiment produced only 3 small-bowel tumours.

The further increase in acid mucins seen among rats receiving jejunoileal bypass in addition to azoxymethane supports our recent finding that goblet cell hyperplasia is a feature of the adaptive response to bypass alone in the colon and jejunoileum remaining in curcuit (Olubuyide *et al.*, 1984). The present studies also confirm our previous contention that subtotal enteric bypass stimulates mucosal growth and promotes chemical carcinogenesis in rat colon (Bristol *et al.*, 1982; 1984). It seems likely that JIB has an enhancing effect on a mucosa already primed by the carcinogen; resection of small intestine has a similar response to bypass (Williamson *et al.*, 1982). The timing of the bypass operation in relation to azoxymethane treatment and the degree of resultant weight loss seem to be critical factors in the development of tumours (Bristol *et al.*, 1982; Williamson, 1980) and may partly explain why colon cancer has not yet been reported after jejunoileal bypass for obesity in man.

Filipe has postulated that an increase in sialomucins, which she found to correlate with the presence of epithelial dysplasia in man and the rat, might represent early malignant transformation of the colonic mucosa (Dawson & Filipe, 1976; Filipe, 1975; Filipe & Branfoot, 1974; Filipe *et al.*, 1982). Our own findings call this hypothesis into question. We have observed similar (though lesser) changes in mucin after jejunoileal bypass alone (Olubuyide *et al.*, 1984), an operation which promotes but does not appear to initiate carcinogenesis (Bristol *et al.*, 1982; 1984; Olubuyide *et al.*, 1984). Moreover, the changes occur throughout the intestinal tract, including the ileum, which is very resistant to carcinogenesis (Williamson *et al.*, 1978; 1980). It seems more likely that a generalised increase in goblet cell numbers is simply one feature of intestinal hyperplasia. Conceivably the bias towards sialomucin production reflects the functional immaturity of epithelial cell in the adapting gut.

We thank Mr P.W. Davies and Mrs C. Williams for technical assistance and the Department of Medical Illustration at Bristol Royal Infirmary for providing the photographs. This work was supported by grants from the Association of Commonwealth Universities of Great Britain, the Cancer Research Campaign, and the South Western Regional Health Authority.

### References

AL-MUKHTAR, M.Y.T., POLAK, J.M., BLOOM, S.R. & WRIGHT, N.A. (1982). The search for appropriate measurements of proliferative and morphological status in studies on intestinal adaptation. In: *Mechanisms of Intestinal Adaptation*. (Eds. Robinson *et al.*), Lancaster: MTP Press Ltd, p. 3.

BRISTOL, J.B., DAVIES, P.W. & WILLIAMSON, R.C.N. (1982). Subtotal jejunoileal bypass enhances experimental colorectal carcinogenesis unless weight reduction is profound. In: *Colonic Carcinogenesis*. (Eds. Malt & Williamson), Lancaster: MTP Press Ltd, p. 275.

BRISTOL, J.B., WELLS, M. & WILLIAMSON, R.C.N. (1984). Adaptation to jejunoileal bypass promotes experimental colorectal carcinogenesis. *Br. J. Surg.*, **71**, 123.

DAWSON, P.A., FILIPE, M.I. (1976). An ultrastructural and histochemical study of the mucous membrane adjacent to and remote from carcinoma of the colon. *Cancer*, **37**, 2388.

FILIPE, M.I. (1975). Mucous secretion in rat colonic mucosa during carcinogenesis induced by dimethylhydrazine. A morphological and histochemical study. *Br. J. Cancer*, **32**, 60.

FILIPE, M.I., BRANFOOT, A.C. (1974). Abnormal patterns of mucus secretion in apparently normal mucosa of large intestine with carcinoma. *Cancer*, **34**, 282.

FILIPE, M.I., SCURR, J.H., ELLIS, H. (1982). Effects of fecal stream in experimental colorectal carcinogenesis. Morphologic and histochemical changes. *Cancer*, **50**, 2859.

GOODLAD, R.A. & WRIGHT, N.A. (1983). Effects of addition of kaolin and cellulose to an elemental diet on intestinal cell proliferation in the mouse. *Br. J. Nutr.*, **50**, 91.

OLUBUYIDE, I.O., WILLIAMSON, R.C.N., BRISTOL, J.B. & READ, A.E. (1984). Goblet cell hyperplasia is a feature of the adaptive response to jejunoileal bypass in rats. *Gut*, **25**, 62.

PEARSE, A.G.E. (1968). *Histochemistry. Theoretical and Applied*. 3rd ed, Vol. 1. London: Churchill.

SHAMSUDDIN, A.K.M. & TRUMP, B.F. (1981). Colon Epithelium II. In vivo studies of colon carcinogenesis. Light microscopic, histochemical and ultrastructural studies of histogenesis of azoxymethane-induced colon carcinomas in Fischer 344 rats. *J. Natl Cancer Inst.*, **66**, 389.

SHAMSUDDIN, A.K.M., WEISS, L., PHELPS, P.C. & TRUMP, B.F. (1981). Colon epithelium. IV. Human colon mucosa adjacent to and remote from carcinomas of the colon. *J. Natl. Cancer Inst.*, **66**, 413.

SPICER, S.S. (1965). Diamine methods for differentiating mucopolysaccharides histochemically. *J. Histochem Cytochem*, **13**, 211.

WILLIAMSON, R.C.N., BAUER, F.L.R., ROSS, J.S., OSCARSON, J.E.A. & MALT, R.A. (1978). Promotion of azoxymethane-induced colonic neoplasia by resection of the proximal small bowel. *Cancer Res.*, **38**, 3212.

WILLIAMSON, R.C.N., BAUER, F.L.R., TERPSTRA, O.T., ROSS, J.S. & MALT, R.A. (1980). Contrasting effects of subtotal enteric bypass, enterectomy and colectomy on azoxymethane-induced intestinal carcinogenesis. *Cancer Res.*, **40**, 538.

WILLIAMSON, R.C.N. (1982). Postoperative adaptation in the aetiology of intestinal cancer. In: *Mechanisms of Intestinal Adaptation*. (Eds. Robinson *et al.*), Lancaster: MTP Press Ltd, p. 621.

WILLIAMSON, R.C.N., DAVIES, P.W., BRISTOL, J.B. & WELLS, M. (1982). Intestinal adaptation and experimental carcinogenesis after partial colectomy. Increased tumour yields are confined to the anastomosis. *Gut*, **23**, 316.